# Incidence of Potential Drug-Drug Interactions in a Limited and Stereotyped Prescription Setting - Comparison of Two Free Online Pharmacopoeias

**DOI:** 10.7759/cureus.886

**Published:** 2016-11-22

**Authors:** Bhaskar Kannan, Amrutha Bindu Nagella, A Sathia Prabhu, Gopalakrishnan M Sasidharan, A S Ramesh, Venkatesh Madhugiri

**Affiliations:** 1 Department of Neurosurgery, Jawaharlal Institute of Postgraduate Medical Education and Research (JIPMER), Puducherry, India.; 2 Department of Anesthesiology and Critical Care, Mahatma Gandhi Medical College and Research Institute, Pondicherry, India; 3 Division of Neurosurgery, Tata Memorial Hospital, Mumbai, India

**Keywords:** drug interactions, adverse drug reactions, epocrates, patient safety, medscape

## Abstract

Background: Drug-drug interactions (DDIs) are very common adverse events in health care delivery settings. The use of electronic pharmacopeias can potentially reduce the incidence of DDIs, but they are often thought to be cumbersome to use. This study is aimed at studying the incidence of potential DDIs in a surgical department, where a limited number of drugs are used in stereotyped combinations. We also compared two popular drug compendia in detecting potential DDIs.

Methods: The prescriptions of selected patients were entered into Epocrates® and Medscape® for Android smartphones. Potential DDIs were generated and their categories were noted. The warnings generated by Epocrates® were compared with those generated by Medscape® and an agreement index was calculated.

Results: Three hundred and thirty-one patients were included for analysis who had received a total of 2,878 drug orders. The incidence of potential DDIs was very high - 89% of all prescriptions. Phenytoin was the drug most commonly implicated, followed by furosemide. Of the DDIs detected, 0.14% were potentially serious and the drug combinations were contraindicated. There was a significant discrepancy between the categories of potential DDIs detected by Epocrates® and Medscape®. No clinically significant DDI was detected in any patient in this cohort.

Conclusions: Despite routinely using only a limited number of drugs in stereotyped combinations, prescriptions in surgical departments may not be immune from a significant incidence of DDIs. The use of free apps could reduce the incidence of DDIs, enhance patient safety, and also aid in educating trainees.

## Introduction

Medication errors are among the most common types of adverse events that occur in health care delivery settings, be it in an inpatient, emergency room, outpatient, or clinic milieu [1]. Such errors account for 16 - 20% of all adverse events in any hospital setting [1-2]. A drug-drug interaction (DDI) is one type of medication error and is a physiological response to a combination of drugs that results in an outcome, which differs from the responses to the agents when administered individually. Some specific and predictable interactions between drugs are exploited, and such drug combinations are used in a deliberate fashion to enhance or modify a therapeutic effect. However, more often, DDIs are an undesirable consequence of pharmacotherapy. DDIs account for nearly 19% of drug-related adverse effects [3]. Most are innocuous and may go unnoticed, but some have the potential to cause significant morbidity. Importantly, the vast majority of DDIs are predictable and preventable.

The implementation of technology, such as electronic medical record (EMR) systems with integrated pharmacopeias and prescription modules, has significantly reduced the number of prescription errors and, consequently, the incidence of DDIs [4-5]. However, these paperless systems are expensive and are not always available in developing countries. This means that the health systems that serve the vast majority of the world’s population do not have access to EMR software with built-in prescription systems.

Several standalone prescribing and drug information databases, which can easily be accessed on portable devices such as smartphones and tablets, have been developed. Several of these databases are free to use and provide not only pharmacologic information regarding drugs but also data regarding possible DDIs for a given combination of drugs.

In this study, we examined the incidence of potential DDIs in a setting with stereotyped prescription practices using two popular digital drug compendia. The first was Epocrates® (Athenahealth, Watertown, MA, USA) since the basic version is free to use and is available on both Android and Apple iOS mobile platforms. We also compared the DDI output data generated by the Epocrates® interaction check module with data generated by the Medscape® (WebMD LLC, NY, USA) interaction check module. Medscape® is a free resource as well.

## Materials and methods

This IRB-approved study (project number JIP/IEC/SC/2014/6/599) was conducted between January to June 2015 (six months) at the Jawaharlal Institute of Postgraduate Medical Education and Research, Pondicherry, India. Exemption from obtaining patient consent was granted (since no direct communication with patients was required for this study) with the caveat that the treating physicians be made aware of every potential warning generated by the apps tested in this study. This caveat was complied with.

All patients admitted by the Department of Neurosurgery during this period were included in this study. Data collected for every patient included demographics, clinical diagnosis at admission, and the list of drugs prescribed. Data pertaining to every patient was included in the study only once, irrespective of the duration of hospitalization and any subsequent changes in the treatment chart. All drugs in a patient's treatment chart were entered into the interaction check module of Epocrates® on an Android smartphone and the potential drug-drug interactions (pDDI) between the drugs prescribed, if any, were noted. The number of interactions, the type of each interaction, and the management strategy advised by the app were recorded. The type of interaction was also classified as pharmacodynamic or pharmacokinetic [6]. The same process for the interaction check was repeated with the Medscape® app. The severity of the pDDI warning was numerically coded on a scale of 0-5 for Epocrates® and 0-3 for Medscape®. Data generated using the two apps was compared, and a coefficient of agreement was calculated. Statistics were performed on Stata (v14.1, College Station, Tx, USA). The treating physician was informed about every potential DDI generated by either app. Every patient was also carefully followed up for any manifestation of a clinically significant drug interaction.

## Results

A total of 331 patients admitted to the Department of Neurosurgery service were included in this study. The mean age of the recruited patients was 39 years; 242 were male and 89 were female (Table 1). The mean number of drugs on the treatment charts was eight (mean = 7.63; SD = 2.75) and a total of 2,878 drug orders had been placed for these 331 patients. There was a total of 89 different drugs prescribed; overall, 1,480 possible pairs were identified that were entered into the interaction check modules of Epocrates® and Medscape®. The numerical codes used to categorize the severity of the pDDIs generated on Epocrates® and Medscape® are listed in Table 2. Interestingly, Medscape® only generated warnings at four levels of severity (m0-m3), whereas Epocrates® had e0-e4. Code e5 on Epocrates® was used to denote a therapeutic advantage. For purposes of comparison, code e3 (Avoid/Use alternative) and e4 (Contraindicated) warnings on Epocrates® were merged and compared with code m3 warnings on Medscape® (Serious - Use alternative) (Table 2).

**Table 1:** Basic Patient Cohort Statistics Basic information regarding the patient cohort recruited for the study.

Mean age of the patients (years)	39.02 (± 17.72)
Sex distribution (M/F)	242/89
Average number of drugs administered	7.63 (± 2.75)
Total number of drugs administered	2,878
Number of unique drugs administered	89

**Table 2:** Categories of Warnings Categories of warnings generated for drug-drug interactions (DDI) on Epocrates® and Medscape®.

Category number	Code used	Category on Epocrates®	N (%)	Code used	Category on Medscape®	N (%)
0	e0	None	41 (2.8)	m0	None	704 (47.6)
1	e1	Caution advised	364 (24.6)	m1	Minor	160 (10.8)
2	e2	Monitor/Modify treatment	888 (60)	m2	Significant - Monitor closely	542 (36.6)
3	e3	Avoid/Use alternative	177 (11.9)	m3	Serious - Use alternative	74 (5)
4	e4	Contraindicated	2 (0.1)	-	-	-
5	e5	Therapeutic advantage	8 (0.5)	-	-	-

### Epocrates®

All 331 patients’ prescriptions were screened for DDIs using the Epocrates® software. In 41 patients’ prescriptions (2.88%), no interactions were noted on Epocrates® and Medscape®. The remaining 295 patients’ prescriptions showed 1,439 potential DDIs (pDDI). These 1,439 DDIs were not generated from unique drug pairs - drug pairs were repeated across patients. Thus, a DDI for one specific pair of drugs may have been listed for many patients if this combination had been prescribed for more than one patient. The total number of interacting drug pairs across the 331 patients’ prescriptions was 1,439. Eight of the 1,439 DDIs were of therapeutic benefit (0.55%). The severity of the undesirable potential interactions detected varied - 0.14% of the drug combinations were advised as being contraindicated (category e4), 11.9% were advised to be avoided (category e3), an alternative drug was suggested and monitoring or modifying therapy was advised in 60% cases (category e2), and caution was advised (category e1) in 24.6% (Table 2). While some of the combinations were administered deliberately to exploit the therapeutic advantage arising out of the additive or synergistic effect (e.g.: Insulin with oral hypoglycemic agents), there was also a risk of additive toxicity and, thus, monitoring was absolutely essential. Phenytoin was implicated most often in the pDDIs, detected by Epocrates®, followed by furosemide and omeprazole (Table 3).

**Table 3:** Commonly Implicated Drugs Ten most common drugs involved in DDIs on Epocrates® and Medscape®.

Drug	Number of DDIs implicated in Epocrates	Number of DDIs implicated in Medscape
Phenytoin	414	375
Furosemide	303	111
Omeprazole	246	99
Ondansetron	193	53
Tramadol	187	105
Dexamethasone	127	111
Pantoprazole	127	5
Mannitol	108	0
Diclofenac	101	101

### Medscape®

There was a significant difference in the pDDIs detected on the Medscape® interaction check module (Table 4). Medscape® detected 776 pDDIs. Thus, nearly half the pairs of drugs listed as having pDDIs by Epocrates® (n = 703, 48.9%) were listed as not having any interactions by Medscape®. The severity distribution of the 776 pDDIs detected by Medscape® was Minor - 10.8% (category m1), Significant, monitor closely - 36.6% (category m2), and Serious, avoid - 5% (category m3). We then separately analyzed the discrepancies between Medscape® and Epocrates®.

**Table 4:** Epocrates® vs Medscape® Crosstabs between the DDI categories generated by Epocrates® and Medscape®. The exact implications of the numerical code is mentioned in Table 2.

	Epocrates® Code						
Medscape Code	e0	e1	e2	e3	e4	e5	Total
m0	41	151	498	4	2	8	704
m1	0	15	122	23	0	0	160
m2	0	191	238	113	0	0	542
m3	0	7	30	37	0	0	74
Total	41	364	888	177	2	8	1,480

The main difference was noted in the pairs where Medscape® listed no pDDI (category m0, n = 704). Of these pairs, Epocrates® listed a potential benefit for eight pairs (category e5), advised caution for 151 (category e1), advised monitoring/modifying treatment in 498 (category e2), advised avoiding the combination in four (category e3), and listed two pairs as absolutely contraindicated (category e4). The Medscape® category 0 pDDIs that generated category 3 or higher pDDIs on Epocrates® are listed in Table 5. No pairs checked were listed as having a therapeutic advantage by Medscape®.

**Table 5:** Analysis of the Discrepancies Analysis of the category 0 pDDIs listed by Medscape® that generated category 3 or higher pDDIs on Epocrates®.

Drug combinations that generated category m0 warnings on Medscape​​®	Epocrates® warning	Category	Risk detailed in Epocrates®
Atenolol + insulin	Avoid/Use alternative	e3	May mask or prolong hypoglycemia
Ondansetron + promethazine	Avoid/Use alternative	e3	May increase risk of QT prolongation and arrhythmias
Glycopyrrolate + potassium chloride	Contraindicated	e4	Contraindicated for solid dosage forms, delays passage of KCL, increased risk of ulcers
Haloperidol + potassium chloride	Contraindicated	e4	Contraindicated for solid dosage forms, delays passage of KCL, increased risk of ulcers

### Unique drug pairs - comparison of the apps

We then examined the drug pairs that had generated the pDDIs on both the platforms. There were 311 unique drug pairs that generated a pDDI either on Epocrates® or Medscape® or on both. Of these, 64.37% were pharmacodynamic in nature, 27.53% pharmacokinetic, and 8.1% of unknown mechanism. The relationship between the categories of warnings generated on Epocrates® and Medscape® for the unique drug pairs are displayed in Table 6. Concordance between the DDI category generated by Epocrates® and Medscape® was seen in 21.3% of the 270 interacting drug pairs. The inter-rater agreement for the categories of pDDIs generated by Epocrates® and Medscape® was assessed by computing Cohen’s kappa. For the data generated by this study, 𝛋 = -0.028 (SE = 0.0326, 95% CI = -0.0920 to 0.0360). This indicates significant disagreement in the pDDI categories generated by Epocrates® and Medscape® [7].

**Table 6:** DDIs for the Unique Drug Pairs The categories of the pDDIs generated by Epocrates® and Medscape® for the 311 unique interacting drug pairs. The categories are the same as listed in Table 2.

	Medscape® DDI Category				
Epocrates® Category	0	1	2	3	Total
0	41	-	-	-	41
1	50	5	45	3	103
2	63	15	43	10	131
3 & 4	5	5	15	9	34
5	2	-	-	-	2
Total	161	25	103	22	311

All the patients were carefully monitored for manifestations of any DDIs. None exhibited signs or symptoms of any DDI.

## Discussion

This study generates data that supports the routine use of an online or digitized pharmacopeia in the absence of an EMR system with an integrated prescription module. The study was conducted in a neurosurgery department where the number of drugs, as well as the combinations used, are potentially stereotyped and limited. For instance, most neurosurgical patients receive anti-seizure medications; in this study, phenytoin was the most commonly employed drug. Most patients with severe cerebral edema receive a combination of mannitol and furosemide. We wished to examine if, in such a setting, the incidence of pDDIs would be less than that reported in the literature, thus, rendering drug pharmacopeias redundant. It was thought likely that in an intensive care unit or outpatient clinic setting, the number of individual drugs prescribed is much higher and, therefore, the potential for DDIs would also be higher. However, this thinking was not borne out by the data we generated. In the present study, 89.12% of patient prescriptions screened showed potential DDIs. On Epocrates®, 1,439 of 1,480 possible drug pairs yielded pDDIs (97.2%). On Medscape®, 52.4% of all possible drug pairs yielded pDDIs. This data is in concordance with a study published by Doubova, et al., which found the incidence of pDDIs to be 80% [8]. In the current study, 0.14% of potential DDIs generated by Epocrates® were category e4 (combinations that were absolutely contraindicated) and 12% were category e3 (avoid/use alternative). On Medscape®, 5% of the drug combinations were category m3, a serious potential for a DDI and using an alternative was recommended. Thus, even in a setting where a limited number of drugs are used and in relatively fixed combinations, between 5-12% of the combinatorial drug pairs demonstrated a potential for major DDIs. Such a setting is, therefore, not immune to the occurrence of clinically significant DDIs.

A potential drug-drug interaction (pDDI) may be defined as an interaction that could theoretically occur between two or more drugs when administered concomitantly [9]. Numerous methods have been devised to calculate the incidence of pDDIs. These include the manual screening of drug prescriptions by clinical pharmacists, screening using drug database software integrated with the EMR systems, or using standalone applications like Epocrates®, Medscape®, etc. Doubova, et al. found the incidence of pDDIs, identified using the Thomson Micromedex® program (Truven Health Analytics, Greenwood Village, CO, USA), to be 80% in ambulatory patients over the age of 50 [8]. A study by Lubinga, et al., which also used Epocrates® to study DDIs, reported an incidence of 23% [6]. Manual evaluation by a clinical pharmacist detected at least one pDDI in the prescriptions of 54% of ICU patients [9]. A similar study on inpatients across all hospital settings found that 19.3% of patients had at least one pDDI [3]. In the primary care setting, a study by Bjerrum, et al. found about 15% of the patients to be at risk of a harmful/serious DDI [10]. Thus, the incidence of pDDIs ranged between 15 to 80%, depending on the clinical setting, the drugs being prescribed, etc. [3, 6, 8-10]. A meta-analysis by Dechanont, et al. found the prevalence rate of DDIs for hospital admissions to be 1.1% and the median DDI prevalence rate for hospital visits was 0.1% [11]. The correlation between the incidence of a pDDI as detected by an app or database and the clinical incidence of an adverse drug reaction is not necessarily a linear one. The incidence of actual clinical DDIs depends on many factors:  the age of the patient, the clinical setting, hepatic and renal function, genetic polymorphisms, etc. [12]. In the present study, despite the high incidence of pDDIs predicted by Epocrates®, no patient actually developed symptoms or signs of a clinically significant DDI. Thus, whereas the incidence of an actual clinically relevant DDI would be dependent on several patient and physiology-related factors, the incidence of pDDIs would only depend on the pharmacology of the drugs. The reason there was such a high incidence of pDDIs in this study was the relatively stereotyped prescription practices in a neurosurgical setting. Thus, a drug pair that generated a pDDI warning would have been repeated for many patients.

An important question would be which drug compendium to employ for routine drug prescriptions. In this study, we compared Epocrates® with Medscape® since the basic versions of both of these platforms are free to use. Both are available as apps on mobile devices and once the app database is fully downloaded, an active internet connection is not required to use the app. The app interfaces are intuitive and easy to understand. These features make the apps comparable in usability. However, in this study, there was a significant difference in the number and types of pDDIs detected by Epocrates® and Medscape® (Tables 4-5). These two databases have been compared head-to-head to evaluate for ease of use and utility in clinical decision-making support, especially for infectious diseases [13-15]. However, to the best of our knowledge, no previous study has compared the categories of pDDIs detected by these apps. We found a significant discrepancy between the warning categories generated by Medscape® and Epocrates®. Large scale studies with clinical correlation would be required to establish which compendium would be the best choice.

The incidence of pDDI alerts would be affected by the method of screening employed. Using highly sensitive software could result in more alerts, which may not necessarily have major clinical implications. This could result in a tendency to ignore or override such warnings. On the other hand, such highly sensitive software would be an important educational tool for medical students and residents. Grading the alerts based on their clinical significance would aid better clinical judgment. The final decision regarding the relevance of an alert and the need to alter therapy would, of course, be at the physician’s discretion. A balance between the sensitivity of detection of DDIs and the clinical implications of these is necessary for effective therapeutic decision making. Larger scale studies would be required to evaluate which app is the best as a pharmacological support system.

We consider it imperative to routinely screen drug prescriptions as this process adds to patient safety to a great extent. Integrating the software with the electronic health record system would be ideal. However, until such time as these systems are universally implemented, stand-alone apps, such as Epocrates® and Medscape®, can be put to effective use. This study was an attempt to establish the usefulness of a stand-alone drug database software in the absence of an electronic health record systems. We have described the incidence, type, and pattern of interactions. These DDIs occur even in settings where a limited and stereotyped prescription pattern exists. We are unable to compute the number needed to treat/harm since no actual adverse clinical events occurred in this study. However, it is important to check every individual patient’s prescription for pDDIs. These software apps can act as a cognitive aid to the physician’s expertise and as an important learning tool for trainees.

## Conclusions

Despite routinely using only a limited number of drugs in stereotyped combinations, prescriptions in surgical departments may not be immune from the significant incidence of DDIs. The use of free apps could reduce the incidence of DDIs, enhance patient safety, and also aid in educating trainees.
